# Human metabolic atlas: an online resource for human metabolism

**DOI:** 10.1093/database/bav068

**Published:** 2015-07-23

**Authors:** Natapol Pornputtapong, Intawat Nookaew, Jens Nielsen

**Affiliations:** Department of Biology and Biological Engineering, Chalmers University of Technology, Göteborg 41269, Sweden

## Abstract

Human tissue-specific genome-scale metabolic models (GEMs) provide comprehensive understanding of human metabolism, which is of great value to the biomedical research community. To make this kind of data easily accessible to the public, we have designed and deployed the human metabolic atlas (HMA) website (http://www.metabolicatlas.org). This online resource provides comprehensive information about human metabolism, including the results of metabolic network analyses. We hope that it can also serve as an information exchange interface for human metabolism knowledge within the research community. The HMA consists of three major components: Repository, Hreed (Human REaction Entities Database) and Atlas. Repository is a collection of GEMs for specific human cell types and human-related microorganisms in SBML (System Biology Markup Language) format. The current release consists of several types of GEMs: a generic human GEM, 82 GEMs for normal cell types, 16 GEMs for different cancer cell types, 2 curated GEMs and 5 GEMs for human gut bacteria. Hreed contains detailed information about biochemical reactions. A web interface for Hreed facilitates an access to the Hreed reaction data, which can be easily retrieved by using specific keywords or names of related genes, proteins, compounds and cross-references. Atlas web interface can be used for visualization of the GEMs collection overlaid on KEGG metabolic pathway maps with a zoom/pan user interface. The HMA is a unique tool for studying human metabolism, ranging in scope from an individual cell, to a specific organ, to the overall human body. This resource is freely available under a Creative Commons Attribution-NonCommercial 4.0 International License.

**Database URL:** http://www.metabolicatlas.org.

## Introduction

Metabolism comprises a large number of biochemical reactions, which are mostly catalyzed by enzymes. Metabolism results in production of essential biochemical compounds and Gibbs free energy to maintain homeostasis of cellular functions. Over the past few decades, more than 2200 human enzymatic reactions (1) have been identified and studied to expand our knowledge of their mechanisms and functions. However, knowledge of each individual enzymatic reaction is not sufficient to obtain an overall picture of human metabolism. Therefore, comprehensive human metabolic models, which provide not only a list of reactions but also relationships between genes and proteins through reactions, are needed for holistic understanding of human metabolism through simulation and data integration.

Genome-scale metabolic models (GEMs) provide comprehensive overview of the genotype to phenotype relationship in living cells and thereby provide a scaffold for interpretation of high throughput data in the context of metabolism (2–4). Metabolism in humans is highly diversified in different cell types. To capture the specific operational mode of metabolism of each human tissue, it is necessary to reconstruct human tissue-specific GEMs (h-tGEMs). Recently, h-tGEMs have been shown to provide much new information about human metabolism when they are integrated with genomic, transcriptomic, proteomic and metabolomic data (5). At present, there are four generic human GEMs publicly available, Recon2 (6) which is updated version of Recon1 (7), the Edinburgh Human Metabolic Network (8), HumanCyc (9) and human metabolic reaction (HMR) (2), which has been updated to HMR2.0, the most comprehensive compilation of HMRs (10), as well as, several h-tGEM (2, 11, 12).

h-tGEMs have been widely used for data analysis, data integration and simulation to gain knowledge about cell type-specific mechanisms prior to whole organism understanding. Knowledge from model-based data analysis has provided new insight about human metabolism, especially in connection with medical research prior to discovery of new treatments or prevention strategies, e.g. to identify new therapeutic agents or novel diagnostic biomarkers (8, 11, 13–15).

Reconstruction of a h-tGEM is generally done by integration of multi-omic data, but organizing several data layers of complex data is a challenge. To deal with integration and organization of biological data for GEM reconstruction, there is a need for a database system that is developed for this particular purpose. In connection with reconstruction of cell type-specific models, we established an HMR database using MySQL (2). The HMR database comprises reactions, related compounds and annotation information. Reaction and compound information in the HMR database was first populated from existing models: the Edinburgh human metabolic network (8) and RECON1 (16) as well as external reaction databases: KEGG (17), HumanCyc (9), BRENDA (18), HMDB (19), ChEBI (20), LMSD (21) and PubChem (22). Annotation data were based on Ensembl (23) and UniProt (24). The next version of HMR (HMR 2.0) incorporated reaction information from RECON2 (6) and HepatoNet (25) that covers many more reactions in an Excel database (10). We soon realized that the extensive coverage of reactions in HMR 2.0 resulted in greater complexity of query commands, greater query processing time and greater complexity of data structures. Data models of both HMR and HMR 2.0 databases were specifically designed for use in conjunction with the (Integrative Network Inference for Tissues) INIT algorithm (2) and expression data from the human protein atlas (26). Furthermore, these databases did not have any user interfaces for easy and efficient query mechanism. Additionally, we also wanted to incorporate other reconstruction methods and more comprehensive network integration, such as evaluation of protein families in comparative genome-scale reconstruction (27) and regulatory information in probabilistic integrative modeling of genome-scale metabolic and regulatory networks (28). To address these limitations and provide enhanced capabilities, we designed and implemented Hreed (Human REaction Entities Database), a new database system (29). Hreed provides flexibility, consistency and crucial features for human metabolism data organization, based on a new database management system (DBMS) concept (29).

To efficiently manage and utilize the h-tGEMs and related information, the human metabolic atlas (HMA) website was built as an online resource to organize and provide comprehensive human metabolic information as models and a database to support further specific analysis or modeling as well as to communicate with the wider research community. There are three major services available on the HMA. The Repository provides management of human GEMs with a crucial feature that enables keeping track of available models. Currently the repository contains 98 human cell type-specific GEMs including models for normal and cancer cell types, 2 curated models and 5 human-related microbial models available to download in system biology markup language (SBML) format for metabolic simulation. To enable exploration of the provided models, a web-based metabolic map visualization system named Atlas, provides a way to explore human tissue-specific metabolic information overlaid on KEGG metabolic pathway maps with an interactive user interface. Finally, the HMA provides Hreed, a reaction database that provides high quality of structured information of metabolic reactions and related data. Future plans include support for cloud-based system and data curation, data expansion and application programming interface (API) for facilitating more advanced capabilities.

## Materials and methods

### Web site and repository development

The HMA website was developed using the Ruby on Rails platform providing basic web application building blocks for the Repository, Hreed and Atlas development. The Repository was developed to provide downloadable sets of GEMs of specific human tissues and related organisms. The models are versioned based on cell types; therefore, the latest release of a specific cell type is the updated version of the same cell type model in the previous release.

### Human REaction Entities Database

#### Database design.

Hreed was designed to organize and maintain the human reaction data represented in the HMA as well as reaction-related data such as genome and annotation information. Due to the large number of tables, and complexity of data relationships used in modeling work, the Structured Query Language (SQL) database described in (2) is highly inefficient for data querying, we decided to develop a noSQL database system, using an object-oriented data model on top of a MongoDB DBMS (refer to http://www.mongodb.org/) that was recently developed (29). The DBMS contains a C++ library with major functions to support data manipulation including file parser, data wrapper and specific database operation functions for data population and integration (29).

#### Data standardization.

The main objectives of the Hreed development were to reduce data redundancy, to maintain data integrity and to improve query efficiency. Uniqueness of data is necessary for integration of multi-level data and to achieve this, all data in the database must be internally consistent, but must also be consistent with external databases with clear identifiers. A systematic data identifier system was therefore developed for compounds, reaction data and cross-references; and all were tightly integrated in the conceptual data layer.

Conventional chemical identifiers are normally specified by names, which provide ambiguous description of chemicals. To provide a unique identifier for chemical compounds, IUPAC developed a unique computer readable identifier of chemical compounds named International Chemical Identifier (InChI) and InChIKey. InChI is comprised several data layers specifically generated from a molecular structure diagram (30). As the number of atoms in a molecule increases, the length of InChI increases and this makes it less suitable as a data identifier. In contrast, the length of InChIKey is constant, namely 25 characters of encrypted InChI string with five information layers. The full description of InChI and InChIKey can be obtained from (30). Provision of InChI and InChIKey is a minimum requirement for storing small molecule data in the Hreed.

One criteria of the Hreed design is to support data integration with other public databases. Different systems of data identifiers, usually only a number, are ambiguous and not suitable for building a rigorous database. To overcome these problems, the MIRIAM registry, the Minimum Information Required in the Annotation of Models registry, was used and also implemented in conceptual data layers. The MIRIAM registry is usually provided as unified resources name (URN) composed of three parts separated by colon sign (:). The first part is prefix, always urn:miriam, to specify the source of registry from MIRIAM. The second part is a name space depended on the data collection of identifier. The last part is the identifier itself. Besides providing global unique identifiers, MIRIAM also provides information for retrieving the original data URL from the original database, which is very useful for avoiding dead links (31).

#### Data population.

Hreed contains several kinds of data including human genes, transcripts, proteins, small molecules and reaction data as shown in Table 1. These are stored in the database as instances of classes corresponding to their tangible biological entities. Genes and transcripts data were automatically populated from Ensembl 69 through the Ensembl biomart service (23). Proteins were from Uniprot release 2012_09 (24). Small molecules and reactions, which are the key data of Hreed, were parsed from HMR (2) including gene–reaction relationship information. However, to support the growth of the human metabolism information in the future, small molecule data were expanded by incorporating an internal compound data pool propagated from HMDB (19), LMSD (21), ChEBI (20), KEGG (17) and PubChem (22) with full InChI annotation.

**Table 1.** Summary of the Hreed data sources

Datasets	Source	Data types	Imported method	Imported/total of original sources
Ensembl gene 69	biomart.org	Gene (and chromosome)	Automatically	62 311
Ensembl transcript 69	biomart.org	Transcript	Automatically	213 272
UniProt 2012_09	uniprot.org	Protein	Automatically	19 084
HMR compound	metabolicatlas.org	Compound	Partial curated	1692/3539
Pooled dataset	metabolicatlas.org	Compound	Automatically	72 594
HMR reaction	metabolicatlas.org	Biological reaction	Partial curated	5282/5526

### Atlas development

The map-rendering engine was developed using a D3 JavaScript (D3JS) library to render metabolic maps from map coordinates into scalable vector graphic (SVG) on a web browser where data can be overlaid. D3JS also provides interactive features to the map. By the virtue of using JavaScript and SVG, Atlas is compatible with mainstream web browsers such as Firefox, Safari, Chrome, Opera and Internet Explorer, without any additional browser add-on requirement.

The map coordinates were taken from KEGG Markup Language (KGML) file, which is the only well-defined data interchange format providing metabolic map with coordinates in a biology-friendly style. These properties of KEGG map facilitate a seamless way to integrate data with the automatic map drawing protocol, and have therefore been widely used for many applications (17). However, KGML is derived from Extensible Markup Language (XML) format that is difficult to parse in JavaScript. To overcome this problem, map information form KGML was imported into newly developed Mjson format. Mjson relies on JavaScript Object Notation (JSON), which is supported in all JavaScript runtime engines.

Atlas has an interactive interface for users to visualize a specific map and retrieved corresponding pathway information by using autocomplete textbox. Pathway list is retrieved directly from KEGG database every time Atlas is opened to ensure that Atlas has the most up to date pathway and map information: cross-references and coordinates. Overlaid information is mapped using related tissue information from HMR2.0 model and Hreed, and gene and cross-reference information retrieved from a KGML file.

## Results

The first release of cell type-specific metabolic models was automatically constructed from a generic GEM and resulted in GEMs for 69 normal and 16 cancer cell types (2). To efficiently manage and utilize information from the reconstructed models, we built the HMA website as an online resource to provide comprehensive information of human metabolism for further specific analysis as well as to provide a user-friendly interface for the research community. The HMA is comprised three parts: Repository, Hreed and Atlas (for visualization).

Besides enabling download of models in SBML format, users can easily access human reaction data from Hreed. Hreed was developed based on an object-oriented data model for storing standardized reactions, compounds and gene annotation information from deposited models. Hreed web interface can be used to easily retrieve reaction data from Hreed, by using specific keywords of related genes, proteins, compounds or cross-references. The query results are downloadable in JSON and Comma Separated Values (CSV) format which is more convenient for further downstream computational analysis. This database query system can also be used for reconstructing user-specified metabolic models.

Atlas metabolic map visualization system provides a comparative view of tissue-specific metabolic networks on KEGG metabolic maps. These data overlaid maps can be used for observing the relationship and differentiation of metabolism for each cell type in a graphical way. The map can be opened by typing into the auto-fill input box or by clicking on the map name on other maps. Summary of the gene identifier for each cell type is shown in a bar chart under the map. Further information can be presented in a popup, upon clicking the gene identifier. To focus on the specific tissues or organ systems, overlaid information can be chosen from a tissue filter tree displayed to the left of the map.

### Human-specific metabolic model repository

The repository is a GEM collection of specific human cells and human-related organisms. The models are publicly available in SBML format. This repository is continuously updated when new GEMs become available. The recent release provides 100 human tissue-specific models including normal, cancer and curated models and five human-related microbial GEMs as shown in Table 2. With the available repositories, the HMA can be considered as a comprehensive web resource for (i) providing draft GEMs for both normal and cancer cell types generated by automatic reconstructions, which can be used as a scaffold for more specialized model reconstruction; and (ii) providing simulation ready models for a few selected cell types and a generic model for human metabolism (HMR2.0), which can be used for simulation of metabolic functions.

**Table 2.** Repository summary

Human tissue-specific model type	INIT normal	INIT cancer	Curated	Human-related microbial
r1 (2)	69	16		
r2 (2)	82			
r3 (15)		5		
r4 (14)			1	
r5 (10)			1	
r6 (32)				5

INIT models are automatically generated GEMs for different human cell types by combining a generic GEM with transcriptomic and proteomic data. The models are continuously generated and updated, triggered by data updates and algorithm improvement updates. The first release models (r1) were generated for 69 different human normal cell types and for 16 human cancer cell types (2), whereas the second release models were generated by a revised INIT algorithm for 82 different human normal cell types (10). In addition, in a study of clear cell renal carcinomas GEMs were generated for five different human cancers (r3) by the revised INIT algorithm (15).

Apart from INIT models, there are two manually curated models deposited in the GEM repository. The Adipocytes1890 model was curated by using 7340 adipocyte-associated genes from immunohistochemistry assays, resulting in a comprehensive and functional GEM (14). Furthermore, to have a functional hepatocyte model for a study of non-alcoholic fatty liver disease, an extensive description of lipid metabolism from HMR2.0 was used for improving the INIT hepatocyte model resulting in iHepatocytes2322 (10).

Several recent studies have shown that the metabolism of the gut microbiome has influence on overall human metabolism, and we therefore also started to collect GEMs for human gut microorganisms. In this version of the HMA, there are GEMs for five key species that are representatives of the human gut microbiome (32). These GEMs provide significant information for understanding the gut microbiome ecosystem as well as its effect on human metabolism.

### Human REaction Entities Database

Hreed is a comprehensive information ecosystem that can support model reconstruction and data curation, and can also serve as a context-aware data query system. This database is an improved version of the HMR database presented earlier (2), and the HMR2.0 database (10). Due to the limitation of data models in the previous database, a new database system, which is designed based on an object-oriented graph data model, was implemented to manage gene, transcript, protein, small molecule and reaction data and to provide high accuracy of human metabolism information to the research community.

**Figure 1.**
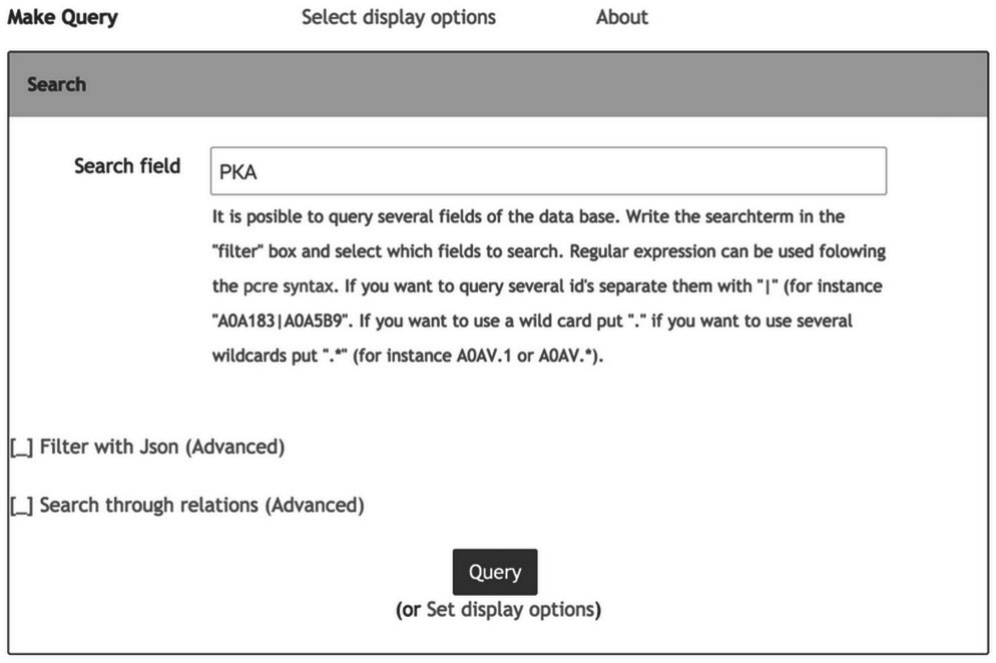
Web-based data query system. Users provide keywords into the filter input box. For complex keywords, ‘|’ and ‘+’ can be used to combine keyword with ‘or’ and ‘and’ operator, respectively.

Information in Hreed can be accessed using the graphical data query interface or the database application programming interface. The graphical data query interface, as shown in Figure 1, is suitable for users without programming knowledge. The data objects can be queried using keywords from their properties such as names, id, cross-references or other object-specific properties including InChI and InChIKey. Query response is in the form of a web page in tablar format, with links to additional details to be pulled from the Hreed itself or from external databases. The query system also provides options for downloading data as a text file in table, XML or JSON format, which is more convenient for further downstream computational analysis.

### Database benchmarking

For benchmarking Hreed, compound and reaction information from the global reconstruction human metabolic model, Recon version 2.02 (6), was used for comparison. Compound data were compared based on InChI, which are provided by both resources. Results in Table 3a show that 74 286 small molecules from Hreed cover more than 93% (910/974) of InChI identified compounds in Recon 2.02. Comparison of reactions from both sources using gene–reaction relationship showed that 1162 reactions with gene association cover about 35% (881/2484) of the reactions in Recon 2.02 as shown in Table 3c.

**Table 3.** Comparison between Hreed and Recon 2.02

(a) Compound	Recon: Compound	Hreed: SmallMolecule
All	5063	74 286
Unique/non-compartment	2626	74 286
With InChI	1332	74 286
With standard InChI	974	74 286

(b) Reaction	Recon: Reaction	Hreed: Reaction

All	7440	2250
Unique/non-compartment	4910	2250
Metabolic reaction	3309	2250
Transport reaction	1601	0

(c) Reaction based on gene association	Recon: Reaction	Hreed: Reaction

All	4252	1162
Unique/non-compartment	2875	1162
Unique metabolic reaction	2484	1162
Transport reaction	391	0

### Atlas: the metabolic map viewer for h-tGEMs

**Figure 2.**
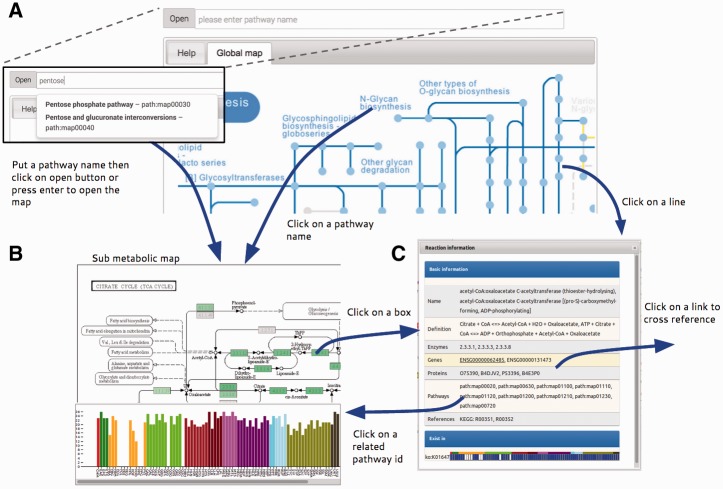
Summary of Atlas map viewer functions. (**A**) The main view of Atlas with control panel comprises a pathway input box for selecting specific sub metabolic map to be opened. The pathway input box provides an autocompleting search by pathway names. Atlas starts with a global metabolic pathway map by default. (**B**) Sub metabolic map with data overlaid and bar plot representing the number of genes that are present in this pathway map for each cell type can be opened by the control panel, clicking on pathway name in every map and clicking on pathway id in the information window. (**C**) The information window represents information of reactions from the KEGG database and provides link to external databases for further information.

To visualize and compare h-tGEMs among different cell types, a visualization system was implemented providing a better view of tissue-specific metabolic models. A web-based visualization system, called Atlas, was developed with an interactive interface representing information from the latest release of INIT normal models on KEGG metabolic maps (17). The Atlas is designed as a web application with an intuitive, clean graphical user interface. The user interface comprises a pathway input box with an ‘Open’ button (a), ‘Tissue filter tree’ (b) and ‘Map viewer’ tabs (c) as shown in Figure 2. Users can easily open a map by typing pathway name or KEGG pathway id, which begins with ‘path:map’ followed by a five digit number in the pathway input box and then click ‘Open’ button or enter. A new map can also be opened by clicking on the corresponding pathway name in a map. To overlay maps with selected tissue information, selecting or deselecting the checkboxes can change the tissue list filter. Only the selected tissue information will be overlaid on maps.

Atlas user interface allows users to interactively explore the map, component details and overlaid data by using simple mouse controls. By mouse scrolling, the user can zoom in and out. Details of map components including id, name, link and graphs of overlaid data will be shown in a balloon popup by a left mouse clicking on the component. By clicking on reactions, usually represented as boxes, a dialog box is displayed, with information of the reaction, related genes, proteins and pathways taken from KEGG and related tissue from the Hreed. Clicking on some text in the dialog box will link directly to major cross-referenced databases, Ensembl (23), UniProt (24), KEGG (17) and Hreed, for further information.

Boxes and lines are shaded in blue. Darker blue means that reaction exists in more tissue-specific model, than ones in lighter blue. Yellow means that reaction does not exist in any tissue-specific model. No color means that reaction does not exist in humans, as per information in KEGG map. Bluish green means that gene exists in corresponded tissue-specific model. Important information is overlaid on the map by using a colorblind-friendly palette suggested by Color Universal Design Organization and http://www.colorbrewer2.org.

## Discussion

The HMA website has been developed as a comprehensive web resource to (i) provide draft h-tGEMs generated by the automatic algorithm INIT, (ii) provide simulation ready functional h-tGEMs which can be used as predictive models and scaffolds for generating personalized GEMs, (iii) provide tools and an environment for further community driven expansion and (iv) provide visualization for comparative analysis of differences in metabolism of specific cell types on a metabolic map.

The direct use of GEMs as a flat file database as well as relational database to provide information of human metabolism is not efficient (2, 33). The Hreed and the Dactyls API library, developed using object-oriented graph data model, can be considered as an innovative tool set for organizing information about human metabolism and advanced application development. The database was specifically designed to support data exchange and enhancement, both by automatic and manual processes. In addition, Hreed includes several data annotation schema such as InChI, InChIKey, reaction key, MIRIAM registry and Open ontology to ensure integrity of the propagated data. With this structure, the database forms a scaffold for driving further annotation of HMRs, in particular in the area of lipid metabolism where many compounds are ill defined and could therefore not be included in Hreed.

Hreed was first propagated from HMR by an automatic script. To achieve the requirements of the database system, propagated information was standardized; especially, small molecules and gene annotation. Only small molecules from HMR that have or can map to InChI identifier were incorporated into Hreed. Also, gene annotations were converted to Ensemble gene id. After a new cycle of model reconstruction, the curated information from GEMs can be integrated back to the database. Due to the flexible data structure, the database can be expanded further for other data categories such as complex molecule, protein–protein interaction and genetic interaction without changing the physical data layer. Besides data structure expansion ability, the database APIs also allow developers to implement new functions, especially data integration and analysis, to the database system in the future.

The Atlas is currently based on KEGG metabolism map, which is easy to automatically render in interactive graphical format in a web browser. To extend the capability of the Atlas, other custom maps from other popular reaction databases: Reactome (34), HumanCyc (9), WikiPathway (35) and probably Panther (36) can be added and incorporated into Hreed in the future, to provide different types of model information.

Compared with the first release of the website containing only a GEM repository, the HMA has been updated with several improvements and new features, and we believe that the HMA web resource will be a valuable data exchange hub for the research community and facilitate new knowledge creation in the field of human metabolism.

## Database availability

The HMA is publicly available on http://www.metabolicatlas.org.
